# Ultimate attainment in L2 semantics: Where and why do learners fail to achieve native-like knowledge of verb meaning?

**DOI:** 10.3389/fpsyg.2026.1719015

**Published:** 2026-04-16

**Authors:** Zhao Akiko Zhao, Yasuhiro Shirai

**Affiliations:** 1 Faculty of Science and Engineering, Waseda University, Tokyo, Japan; 2 Department of Cognitive Science, Case Western Reserve University, Cleveland, OH, United States

**Keywords:** adult second language acquisition, collocation learning, critical period, semantic knowledge, ultimate attainment

## Abstract

This study investigates the development of L2 lexical semantics in advanced Chinese learners of Japanese, focusing on the role of length of residence (LOR). Differences in semantic domains between learners’ first language (L1) and second language (L2) often hinder accurate word meaning acquisition. Cross-linguistic differences between L1 and L2 are particularly evident in verb–noun collocations, which pose persistent challenges even for advanced learners. Sixty-six Chinese learners of Japanese with varied lengths of residency and 32 native Japanese speakers (NSs) participated in an acceptability judgment task involving the following three types of collocations: (1) L1-only collocations (e.g., *eakon-o akeru ‘open the air conditioning’), which are valid in Chinese but not in Japanese; (2) L2-only collocations (e.g., *eakon-o tsukeru* ‘turn on the air conditioning’), which are valid only in Japanese; and (3) L1-L2 collocations (e.g., *doa-o akeru* ‘open the door’), which are valid in both languages. The results revealed that while NSs as a group rated L1-only collocations as unacceptable and L2-only collocations as acceptable, medium-LOR learners (3–7 years of residence) showed non-target-like patterns across the three collocation types. Long-LOR learners (more than 10 years) exhibited native-like judgments for L2-only collocations but continued to differ from NSs in their acceptance of L1-only collocations. Overall, these findings suggest that extended residence facilitates partial lexical-semantic integration in L2 achieved via positive evidence in the input, while persistent L1-mediated representations may constrain ultimate attainment in lexical-semantic development, which may persist due to a lack of negative evidence.

## 1 Introduction

Adult second language (L2) learners often rely on native-language (L1) meanings when acquiring new L2 vocabulary. Although this L1-based mapping supports early learning, it can hinder the development of native-like semantic boundaries, especially when L1 and L2 differ in terms of how they categorize meanings (Tanaka and Abe, 1985; Saji and Imai, 2013; Wolter and Yamashita, 2015, 2018). However, despite extensive research on L2 acquisition, studies on ultimate attainment have focused primarily on morphosyntax, revealing that some adult learners reach near-native proficiency in certain syntactic domains (e.g., Birdsong, 1992; Kellerman, 1995) but often continue to diverge from native speakers in terms of morphology (Lardiere, 2007). In contrast, far less is known about whether adult learners can achieve native-like attainment in lexical semantics—particularly in collocational knowledge, which is highly sensitive to L1 influence (Yamashita and Jiang, 2010).

Research on semantic development has focused primarily on phrasal-level semantics or syntax–semantics interfaces (e.g., Slabakova, 2006), leaving a critical gap regarding the ultimate attainment of lexical-semantic knowledge—specifically, the acquisition of collocational patterns that differ across languages. Cross-linguistic divergences in lexicalization illustrate this challenge. For example, the Chinese verb *kai* ‘to open’ applies broadly to both physical objects and abstract events (e.g., *kai men* ‘open the door’, *kai deng* ‘turn on the light’, *kai ye* ‘open for business’, and *kai xue* ‘start school’), whereas other languages (e.g., English and Japanese) rely on distinct verbs in these contexts. Such semantic mismatches may lead even highly proficient learners to overgeneralize or misinterpret verb–object combinations in the L2.

Thus, the central question that remains unaddressed is whether adult L2 learners—particularly those with long lengths of residence and near-native overall proficiency—can achieve native-like collocational knowledge in semantic domains where L1–L2 mappings diverge. Little is known about how such learners internalize the boundaries of L2 word meanings or whether L1-based conceptual structures continue to shape their judgments.

The present study addresses this gap by examining medium-length-of-residence (LOR) learners (three to 7 years of residence) and long-LOR learners (more than 10 years of residence). We investigate how L1–L2 semantic mapping influences learners’ collocational judgments and assess whether this influence persists even among learners who have nearly reached ultimate proficiency. By focusing on lexical-semantic attainment in contexts involving divergent semantic domains, the present study attempts to advance our understanding of whether adult learners can fully acquire L2 lexical semantics and to identify the factors—such as transfer from L1—that may limit or facilitate such attainment.

## 2 Literature review

### 2.1 Ultimate attainment in the acquisition of L2 syntactic and morphological ability

The question of whether adult L2 learners can attain native-like proficiency has been a central issue in L2 acquisition research (Kellerman, 1995; Lardiere, 2007; Shirahata, 2006). A number of studies have reported that near-native proficiency is possible, particularly in the domains of syntax and morphology (White and Genesee, 1996; Lardiere, 2007; Abrahamsson and Hyltenstam, 2009; Hopp, 2010; Ionin and Montrul, 2010).

One finding that has consistently been reported in these studies pertains to the role played by the LOR in the target language environment. Flege et al. (1999), for example, reported that both age of acquisition (AoA) and LOR influence learners’ proficiency in terms of pronunciation and morphosyntax. Although phonology is not the focus of the present study, it is worth noting that phonological proficiency is also characterized by a strong LOR effect. Granena and Long (2013), who investigated native Chinese speakers learning Spanish, demonstrated that more than 10 years of exposure was associated with higher levels of ultimate attainment in the contexts of phonology, lexis, and morphosyntax. Similarly, Shirahata (2006) reported that Japanese L2 learners who have lived in Japan for more than 10 years judge reflexive pronouns in the same manner as do native speakers (NSs), whereas those who have had only 5 years of residency do not. White and Genesee (1996) also reported native-like performance among L2 learners with more than 10 years of immersion in a grammaticality judgment task focusing on WH questions.

Overall, the findings presented above indicate that extended exposure—specifically, more than 10 years of exposure—can serve as a necessary condition for native-like attainment in various linguistic domains. However, most of these studies have focused on morphosyntactic or phonological abilities, and much less is known regarding the ultimate attainment of semantic knowledge in L2. The present study addresses the issue of semantics with the aim of investigating whether similar patterns of attainment are evident in this underexplored domain.

### 2.2 The effects of AoA and ultimate attainment in semantics

No overarching consensus has been reached regarding the ultimate attainment of semantic knowledge in the context of L2 learning (see Long, 1990; Yorio, 1989 for early discussions of this topic). Slabakova (2006), however, reviewed neurophysiological studies and concluded that no critical period for phrasal semantics—which pertains to the meaning derived from syntactic units larger than individual words, such as phrases or sentences—and no barrier to achieving native-level semantics can be observed, thus suggesting that adult L2 learners can reach this level. Studies that have used neurofunctional imaging (PET, fMRI) and electrophysiology (ERP) have indicated that semantic integration is processed similarly by both NSs and highly proficient L2 learners, whereas syntactic and morphological processing—such as inflectional morphology, gender, or case—depends more heavily on the AoA than does semantic processing. For example, Hahne (2001) used ERP to reveal that while semantic violation (e.g., #*Der Ozean wurde geschlossen*, “The ocean was being closed”) is processed similarly by NSs and L2 learners, syntactic violations (e.g., *Das Geschäft wurde am geschlossen, “The shop was being on closed”) are not^1^. These findings suggest that adult L2 learners can achieve native-level semantic proficiency, according to Slabakova (2006).

While Slabakova (2006) offered important insights into ultimate attainment in the context of adult L2 semantic knowledge, her work did not specifically address lexical semantics, which pertains to the meanings of individual words and phenomena such as polysemy. For example, the Chinese verb *kai* (开) ‘open’ has multiple related meanings, including ‘to open a door’ (开门), ‘to turn on a light’ (开灯), ‘to drive’ (开车), ‘open for business’ (开业), and ‘to start school’ (开学). Previous studies have reported that such semantic nuances often entail challenges for L2 learners and can lead to persistent differences between NSs and L2 users (e.g., Tanaka and Abe, 1985; Malt and Sloman, 2003; Wolter and Gyllstad, 2011, 2013; Saji and Imai, 2013).

Overall, previous studies on ultimate attainment in second language acquisition suggest that long-term immersion in an L2-speaking environment contributes to stable and highly advanced proficiency, as demonstrated in research on morphosyntactic development (e.g., Birdsong, 1992; White and Genesee, 1996; Shirahata, 2006; Abrahamsson and Hyltenstam, 2009; Hopp, 2010) and phonological attainment (e.g., Granena and Long, 2013). In contrast, relatively few studies have examined ultimate attainment in the domain of semantic ability using neurophysiological methods, and such studies, employing electrophysiological and neuroimaging techniques (e.g., Weber-Fox and Neville, 1996; Hahne, 2001; Rossi et al., 2006), have focused primarily on phrasal semantic processing (Jackendoff, 2002: pp. 378–421) rather than on word-level lexical semantics. These studies have shown that late L2 learners can process phrasal semantic information in a manner comparable to native speakers, a finding that has been interpreted as evidence that native-like semantic attainment is possible even when L2 acquisition begins in adulthood (Slabakova, 2006). At the same time, the relative paucity of research on lexical semantics leaves open the question of whether similar levels of attainment can be achieved for word-level meaning representations, particularly in cases involving polysemy and subtle semantic distinctions.

### 2.3 Word learning by adult L2 learners in input-rich and input-poor environments

Although the ultimate attainment of lexical-semantic knowledge in L2 learners has not been investigated thoroughly, previous studies on this topic have suggested that medium-LOR learners often exhibit evidence of the influence of L1 and fail to attain native-like lexical semantic competence. These learners tend to accept word meanings that differ from the norms observed among NSs, thus reflecting the reliance of the former on interpretations that are rooted in their native language (Tanaka and Abe, 1985; Jiang, 2000; Wolter and Gyllstad, 2011; Wolter and Yamashita, 2018; Saji et al., 2024).

Previous research has also explored how different learning environments—namely, input-rich versus input-poor contexts—affect the acquisition of lexical semantic knowledge. For example, Saji and Imai (2013) investigated Japanese learners of Chinese who studied in China (i.e., an input-rich environment) for 2–3 years, whereas Wolter and Yamashita (2015, 2018) examined Japanese learners of English who received only classroom instruction in Japan (i.e., an input-poor environment). Despite the differences in input availability, both groups exhibited difficulties in the context of processing L2-specific words and collocations that lack direct equivalents in their L1, as well as L1-specific expressions that lack clear counterparts in L2 (Wolter and Gyllstad, 2011; Wolter and Yamashita, 2015, 2018).

In the context of a primed lexical decision task, Wolter and Yamashita (2018) reported that Japanese university students learning English in Japan (i.e., an input-poor environment) struggled in particular with incongruent collocations — namely, expressions that are acceptable in one language but not in the other, including L2-only collocations (e.g., *bad debt*, not possible in Japanese, *warui syakkin) and L1-only collocations (e.g., *katsudoutekina seikaku ‘active personality’*, acceptable in Japanese but not in English). Furthermore, this difficulty was observed to be greater in the context of L1-only collocations. Although Wolter and Yamashita’s study did not offer a conclusive explanation for this asymmetry, it highlighted the persistent influence of L1 semantic structures. Furthermore, learners attained better performance with respect to high-frequency collocations than with regard to low-frequency collocations, thus highlighting the critical role played by input frequency in collocational knowledge (Wolter and Gyllstad, 2013).

Similarly, Saji and Imai (2013) reported that even learners who have been immersed in an input-rich environment for 2–3 years continue to exhibit a strong influence of L1 in terms of their interpretation of Chinese word meanings, despite their ability to handle university-level content. A more recent study conducted by Saji et al. (2024) examined Chinese NSs learning Japanese in an input-poor environment (a university in China). In the context of a verb production task that was based on videos of cutting and breaking events, participants selected Japanese verbs such as *kiru* ‘cut’, *waru* ‘split’, *saku* ‘tear’, *oru* ‘fold’, *yaburu* ‘tear’, and *chigiru* ‘tear (by hand)’—verbs that all translate as approximately to ‘cut’ or ‘break’ in English but differ in terms of their fine-grained semantic boundaries. The L2 learners’ verb choices diverged significantly from those of native Japanese speakers, thus indicating persistent L1-based interpretations of meaning.

Saji et al. (2024) argued that L1 transfer is particularly strong during the early stages of L2 word learning, and although this influence may evolve as proficiency increases (Jiang, 2000), negative transfer can persist in later stages. To acquire native-like sensitivity to semantic boundaries, extensive and varied inputs are essential (Saji and Imai, 2013; Saji et al., 2024).

However, the question of whether 2–3 years of learning—regardless of the input environment—are sufficient to facilitate the achievement of native-like knowledge in lexical semantics remains unanswered. It is clear from Wolter and Yamashita (2018) that learners who have undergone more than 3 years of exposure have not been adequately investigated in this aspect of L2 lexical-semantic acquisition. Since learners who have undergone more than 10 years of exposure to L2-speaking environments have been reported in previous studies to achieve morphosyntactic attainment approaching native-like competence, it is important to investigate whether native-like lexical-semantic competence is attainable. The present study thus includes learners who have received extended exposure—especially those who had more than a decade of LOR—with the aim of investigating ultimate attainment in the context of L2 lexical semantics, a domain that remains largely unexplored.

### 2.4 Developmental changes in L2 word learning

**Figure 1:**
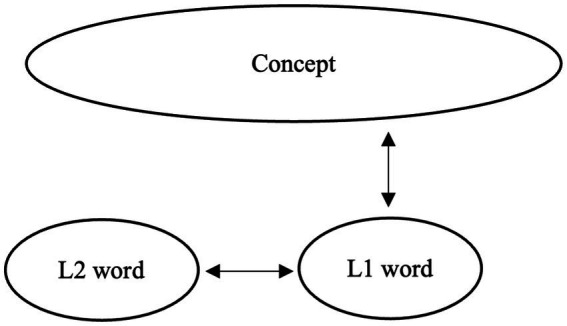
Image of L2 word learning: The first stage.

**Figure 2:**
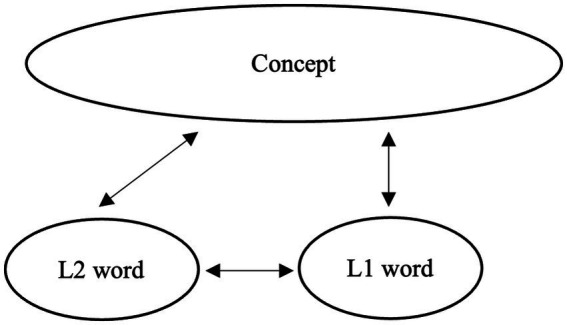
Image of L2 word learning: The second stage.

**Figure 3:**
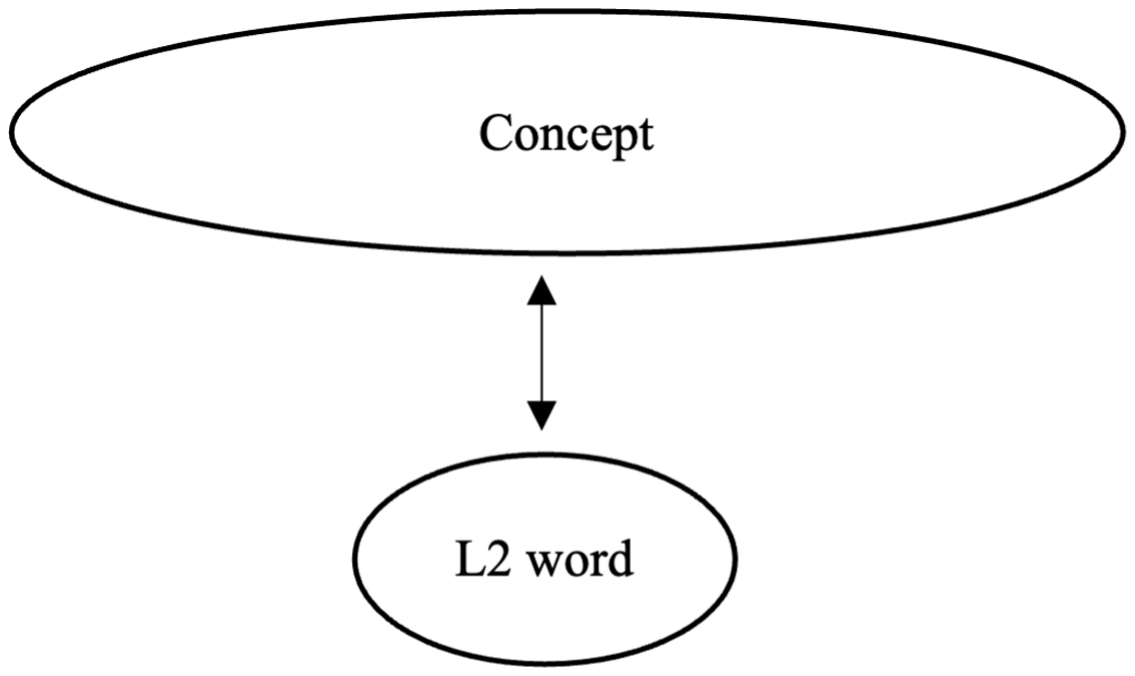
Image of L2 word learning: The third stage.

Understanding word meaning is a central component of semantic competence. According to Jiang (2000), the development of lexical knowledge in a second language (L2) proceeds through three stages, during which connections between L2 lexical forms and their conceptual representations are gradually established. In the initial formal stage, learners create L2 lexical entries that contain only surface-level information, such as phonological and orthographic properties, without associated semantic content. In the subsequent lemma mediation stage, semantic and syntactic information from the first language (L1) translation equivalent is transferred to the L2 lexical entry, such that L2 word use is mediated by embedded L1 lemma information and by associative links to L1 forms. Finally, in the L2 integration stage, semantic, syntactic, and morphological features become fully incorporated into the L2 lexical entry. At this stage, lexical representations are assumed to be functionally equivalent to those of native speakers (NSs) in terms of both structure and processing (Jiang, 2000, p. 53; see Figures 1–3).

While Jiang’s model was originally proposed to account for the acquisition of individual lexical items, it also provides a useful framework for understanding the development of collocational knowledge, which inherently involves constraints extending beyond word meaning. Collocations require learners to integrate semantic compatibility, argument structure, verb–noun selection restrictions, and fixed morphological forms (e.g., tense or number). Consequently, native-like collocational use presupposes not only knowledge of individual word meanings but also grammatical precision, frequency-sensitive familiarity, and contextual appropriateness. For this reason, the L2 integration stage, which entails the full integration of semantic, syntactic, and morphological information, is particularly relevant for explaining how L2 learners come to acquire collocations in native-like ways.

Previous studies suggest that many advanced L2 learners may remain at the lemma mediation stage, especially in domains where L1 and L2 differ in their collocational patterns. For example, learners examined in prior research on lexical semantics and collocations (e.g., Wolter and Yamashita, 2018) often exhibit collocational judgments that reflect L1-based associations and incomplete lexical restructuring. In particular, collocations that are acceptable in the L1 but not in the L2 (L1-only collocations) appear to be especially susceptible to persistent L1 mediation. Yamashita and Jiang (2010) similarly reported that even advanced learners show reduced accuracy and slower processing for incongruent collocations, suggesting that traces of L1 influence may persist when collocational mappings diverge across languages. However, because the learners in these studies had relatively limited lengths of L2 exposure, it remains unclear whether extended exposure leads to further lexical integration, particularly for collocations that lack direct L1 equivalents.

Building on Jiang’s model, the present study examines persistent L1 mediation and incomplete lexical integration as mechanisms underlying non-native-like collocational knowledge. Specifically, we investigate how different collocation types map onto different developmental stages. L1-only collocations are predicted to elicit higher acceptability ratings from L2 learners than from native speakers, reflecting persistent reliance on L1-based semantic representations; critically, such over acceptance is expected to persist even among learners with long LOR of 10+ years. In contrast, L2-only collocations, which lack direct L1 equivalents, are not constrained by competing L1-based representations but instead lack support from L1-based semantic scaffolding. As a result, their acquisition is expected to be strongly influenced by the consistency of L2 input rather than by cross-linguistic transfer. As learners accumulate exposure, repeated encounters with these collocations facilitate the strengthening of L2-specific form–meaning mappings, leading to increasingly target-like acceptability judgments with increased LOR. However, if lexical integration remains incomplete, then residual differences from native speakers may still remain. Finally, collocations shared across L1 and L2 are predicted to be acquired earlier than are collocations that are unique to either the L1 or the L2 (i.e., L1-only and L2-only collocations) and to exhibit smaller group differences than both L1-only or L2-only collocations across learner groups. By examining these patterns across learners with different LORs, the present study seeks to clarify whether extended exposure enables adult learners to reach the integration stage for collocational knowledge or whether L1-based mediation continues to constrain ultimate attainment.

### 2.5 The current study

Previous research on ultimate attainment in adult second language (L2) acquisition has focused primarily on formal linguistic domains, particularly syntax and morphology. Studies have consistently shown that with long-term residence in a target language environment—often operationalized as approximately 10 years or more—adult L2 learners can attain near-native levels in syntactic competence, approaching native speakers (NSs) in terms of grammatical judgments and processing (e.g., Johnson and Newport, 1989; Shirahata, 2006; Ionin and Montrul, 2010). In contrast, morphological accuracy has been reported to remain more vulnerable, even among highly proficient long-term residents, suggesting persistent difficulty in this domain (e.g., Lardiere, 2007).

Although ultimate attainment in morphosyntactic domains has been examined extensively, this body of research has focused predominantly on syntax, with morphology often shown to lag behind, even at advanced stages. In contrast, considerably less attention has been paid to the ultimate attainment of semantic knowledge. Existing studies on L2 semantics have concentrated largely on phrasal or construction-level meanings, such as verb–argument mappings or event representations (e.g., Saji and Imai, 2013), rather than on the fine-grained structure of lexical semantic representations. In particular, when lexical semantic boundaries differ between learners’ first language (L1) and the L2, learners often rely on L1-based semantic mappings, leading to non-target-like acceptability judgments, especially for collocations that are felicitous only in the L1 (e.g., Wolter and Yamashita, 2018).

Crucially, whether such L1-mediated lexical semantic representations persist even after extended exposure to the L2 remains unclear. To date, few studies have examined the semantic competence of learners with more than 10 years of residence—an exposure length often associated with ultimate attainment in syntax (e.g., Granena and Long, 2013).

The present study addresses this gap by investigating lexical-semantic representations in highly proficient Chinese-L1 learners of Japanese with different lengths of residence (LOR). Specifically, we compare learners who have lived in Japan for more than 10 years (long-LOR) with those who have lived there for 3–7 years (medium-LOR), as well as with Japanese NSs. By examining acceptability judgments of L1-only, L2-only, and L1–L2 shared collocations, this study aims to clarify whether extended exposure leads to native-like lexical semantic representations or whether L1 influence persists even at advanced stages of L2 acquisition. Based on the theoretical framework and previous findings, the present study addresses the following research questions:

*RQ1*: Do Chinese learners of Japanese with different lengths of residence (LOR) differ in the target-likeness of their acceptability ratings of Japanese collocations, and how do both learner groups compare with native speakers of Japanese?

*RQ2*: Does collocation type (L1-only, L2-only, and L1–L2 shared) modulate acceptability judgments differently across learner groups and native speakers?

On the basis of previous research and theoretical models of L2 lexical development, the present study advances the following hypotheses:

*Hypothesis 1*: Acceptability judgments are expected to differ systematically across groups as a function of length of residence (LOR), as indexed by learners’ accuracy relative to native-speaker judgments. Specifically, long-LOR learners are predicted to show higher accuracy in acceptability judgments than are medium-LOR learners. However, even long-LOR learners are expected to differ from native speakers overall, indicating incomplete convergence in lexical-semantic representations.

*Hypothesis 2*: The effect of collocation type on acceptability judgments is expected to reflect differential degrees of L1 mediation, as indexed by learners’ convergence with or divergence from native-speaker judgments. In particular, incongruent collocations (i.e., L1-only and L2-only collocations) are expected to yield greater divergence from native-speaker judgments than congruent collocations (i.e., shared L1–L2 collocations). Specifically, for L1-only collocations, both medium- and long-LOR learners are predicted to differ from native speakers, indicating persistent L1 influence. In contrast, for L2-only collocations, medium-LOR learners are expected to differ from native speakers, whereas long-LOR learners are predicted to show native-like performance. For shared L1–L2 collocations, no systematic differences between learners and native speakers are expected across LOR groups.

## 3 Methods

The present study investigates whether long-LOR learners and medium-LOR learners differ in terms of their judgments of L1–L2, L2-only and L1-only collocations. For example, in the context of expressing the meaning ‘to close the door’, *guan* is used as the verb in Chinese (*guan deng* ‘close the door’), whereas *shimeru* ‘close’ is used in Japanese (*doa-o*^2^ *shimeru* ‘close the door’), both of which convey the meaning ‘to close’ and are considered intuitively by bilingual individuals to be direct translations of one another. However, the Chinese *guan* ‘close’ can also collocate with object NPs such as lights, air conditioning and computers, whereas its Japanese lexical equivalent *shimeru* ‘close’ cannot; instead, *kesu* ‘turn off’ should be used in these contexts because it indicates stopping the operation or flow of something that is in a state of activation (*eakon-o kesu* ‘turn off the air conditioner’). Thus, comparisons of judgments such as *denki-o shimeru ‘close the light’ (L1-only collocation), *denki-o kesu* ‘turn off the light’ (L2-only collocation) and *doa-o shimeru* ‘close the door’ (L1–L2 collocation) would reveal the learning of L2-specific semantic knowledge and the interference of L1 translation equivalents (Table 1).

**Table 1:** Examples of target verbs in Japanese and their Chinese equivalents.

	Semantic domain in Japanese	Semantic domain in Chinese	L1–L2 collocation	L2-only collocation	L1-only collocation
akeru (to open)	tsukeru (to turn on)	kai (to open, to turn on)	JP: *doa-o akeru* CN: *kai men* ‘Open the door’	JP: *ana-o akeru* CN: *kai dong ‘Open a hole’	JP: *eakon-o akeru CN: *kai kong tiao* ‘Open the air-conditioning’
shimeru (to close)	shimeru (to close)	guan (to close, to turn off)	JP: *mado-o shimeru* CN: *kai chuang zi* ‘Close the window’	JP: *futa-o shimeru* CN: *guan gai zi ‘Close the bottle’	JP: *terebi-o shimeru CN: *guan dian shi* ‘Close the television’
taberu (to eat)	taberu (to eat)	chi (to eat, to drink)	JP: *pan-o taberu* CN: *chi mian bao* ‘Eat bread’	JP: *okayu-o taberu* CN: *chi xi fan ‘Eat porridge’	JP:*kusuri-o taberu CN: *chi yao* ‘Eat medicine’
nomu (to drink)	nomu (to drink)	he (to drink, to eat)	JP: *juusu-o nomu* CN: *he guo zhi* ‘Drink juice’	JP: *kusuri-o nomu* CN: *he yao ‘Take medicine’	JP: *okayu-o nomu CN: *he zhou* ‘Drink porridge’
sumu (to live)	sumu (to live)	zhu (to live, to stay)	JP: *tokai-ni sumu* CN: *zhu cheng shi* ‘Live in a city’	JP: *yado-ni tomaru* CN: *zhu zhu su ‘Stay in a hut’	JP:*hoteru-ni sumu CN: *zhu jiu dian* ‘Live in a hotel’
tsukuru (to make)	tsukuru (to make)	zuo (to make, to do) you (to have)	JP:*ryouri-o tsukuru* CN: *zuo fan* ‘Make a food’	JP: *omoide-o tsukuru* CN: *zuo hui yi ‘Make a memory’	JP:*yume-o tsukuru CN: *zuo meng* ‘Make a dream’
agaru (to go up)	agaru (to go up, to enter someone’s house)	shang (go up, to take, to enter)	JP: *butai-ni agaru* CN: shang wu tai ‘Go up a stage’	JP: *wadai-ni agaru* CN: *shang hua ti ‘Go up trending topic’	JP:*denshya-ni agaru CN: *shang dian che* ‘Go up on a train’

### 3.1 Participants

Sixty-six native Chinese speakers who were learning Japanese as an L2 (non-native speakers, i.e., NNSs) and 32 Japanese NSs who lived in Japan participated in this study. In the present study, the NNSs were separated into two groups depending on their LOR in the country in which the target language is spoken, i.e., Japan. Thirty-three participants (medium-LOR learners) had been living in Japan for 3–7 years, and 33 participants (long-LOR learners) had been living in Japan for more than 10 years. A significant difference was observed in the LOR in Japan between long-LOR learners (*M* = 13.1 years; *range* = 10–21) and medium-LOR learners (*M* = 5.0 years; *range* = 3–7) (*t* = 14.78, *df* = 32, *p* < 0.001). All L2 learners had attained the highest level (N1) on the Japanese Language Proficiency Test (JLPT) during their first 2 years in Japan. The highest level of the JPLT (i.e., N1) corresponds to level C1 in the Common European Framework of Reference for Languages (CEFR)^3^. Table 2 provides detailed information concerning the participants. All of the participants used Japanese in their daily lives, particularly in their work or studies. The participants’ ages ranged from 19 to 45 years, and they were recruited from the Tokyo area.

**Table 2:** Participant information.

Group	N	Age	Sex (M/F)	Age at the start of residence in Japan	Length of residence in Japan
NS	32	33.5	9/23	–	–
NNS_ Long-LOR	30	34.7	11/22	21.3	13.3
NNS_Medium-LOR	32	26.6	14/19	21.5	5.2

Participants were prospectively recruited between April 2020 and March 2021. All participants were adult Chinese residents living in Japan. Written informed consent was obtained from all participants prior to participation. Participation was voluntary, and participants could withdraw at any time without penalty.

### 3.2 Materials

The study used a set of 90 Japanese sentences featuring target collocations composed of specific verb–noun combinations. The selection of these collocations followed a multistep procedure combining expert judgment and empirical verification. First, a pool of candidate verb–noun combinations was compiled by the first author, who is bilingual in Mandarin Chinese and Japanese, in collaboration with a bilingual Japanese-language teacher and a bilingual Chinese-language teacher. Based on their professional and pedagogical experience, the candidate items were chosen to reflect collocations that bilingual learners are known to confuse or misuse, often due to cross-linguistic influence.

The candidate collocations were then classified into the following three categories: L1-only, L2-only, and shared L1–L2 collocations (see Table 1 for examples). To validate the choice of items, corpus-based frequency checks were conducted. For L2-only and shared L1–L2 collocations, their frequencies of occurrence were examined in large-scale Japanese corpora (Tsukuba Web Corpus^4^). L1-only collocations were confirmed to occur in Chinese but to be absent or extremely rare in Japanese; when they appeared in Japanese, their use was restricted to marked or non-target contexts (e.g., metaphorical or device-specific interpretations). In addition, the resulting stimulus set was evaluated by three native speakers of Mandarin Chinese and three native speakers of Japanese, who were different from the speakers consulted during the item construction phase. The evaluators independently judged both the naturalness of each collocation and its collocation category membership. For each item, agreement was calculated as the proportion of evaluators assigning the same judgment. Only items for which at least 90% of judgments were consistent across evaluators (i.e., agreement by at least 27 out of 30 judgments) were retained for inclusion in the final materials.

The final stimulus set consisted of 90 test sentences, with 30 sentences in each collocation category. L1-only collocations (e.g., *eakon-o akeru ‘open the air conditioning’ or *hoteru-ni sumu ‘live in the hotel’) are acceptable in L1 (Chinese) but not in the L2 (Japanese). L2-only collocations (e.g., *eakon-o tsukeru* ‘turn on the air conditioning’ or *hoteru-ni tomaru* ‘stay in the hotel’) are acceptable in the L2 but not in the L1. Shared L1–L2 collocations (e.g., *doa-o akeru* ‘open the door’ or *manshon-ni sumu* ‘live in a condo’) are acceptable in both languages. The complete list of test sentences is provided in the Appendix.

All acceptable collocations included in the materials (i.e., L2-only and shared L1–L2 collocations) had frequencies exceeding 10 in the Tsukuba Web Corpus (see Footnote 3), which contains approximately one billion words drawn from Japanese-language websites. The frequency range for L2-only collocations was 11–9,008 (*N* = 1,630, *M* = 621, SD = 1,658), whereas that for shared L1–L2 collocations was 19–13,148 (*N* = 2,534, *M* = 1,567, SD = 2,578).

### 3.3 Procedure

The participants were instructed to judge whether the 90 sentences explained in the Materials section were acceptable in Japanese on a four-point scale (1 = unacceptable; 2 = slightly unacceptable; 3 = slightly acceptable; 4 = acceptable)^5^. The survey was conducted via a Microsoft Word file, which the participants received by email. The test items were presented in a randomized order. The three collocation types (L1-only, L2-only, and L1–L2 collocations) were counterbalanced across participants such that no collocation type appeared disproportionately early or late in the survey. This procedure was intended to ensure that potential order effects would be minimized.

Participants were instructed to complete the questionnaire independently in a quiet environment, such as their room, library, or office. According to their self-reports, the task required approximately 30–40 min to complete. To ensure that they completed the task under comparable conditions, they we instructed to complete the task in one sitting, and not to consult dictionaries or online resources. Participants were informed that the task would typically take approximately 30–40 min, which served as a general guideline rather than a strict time limit. After data collection, response patterns were inspected to identify potential anomalies (e.g., invariant response patterns, which suggests mechanical choices by participants); no participants were excluded on this basis.

To elicit judgments that reflected lexical naturalness rather than purely focusing on grammaticality or context-dependent interpretation, we intentionally chose not to present collocations in isolation. Some verb–noun combinations may be acceptable in specific contexts but not in that of the target meaning that we aimed to test. For example, while “eakon-o akeru” (‘open the air conditioner’) is generally considered to be anomalous in Japanese, it may be acceptable in contexts such as opening the front panel of the device during cleaning. To control for such contextual effects, each collocation was embedded in a short, neutral sentence. The target collocation within each sentence was highlighted visually through the use of square brackets (【 】), as shown below, although actual items are presented in normal Japanese orthography (with Chinese and kana charachters, and without English translation, of course).

*Totemo atsui node eakon o 【akete】 heya no ondo o sagemasu* (“It is very hot, so we will 【open】 the air conditioner to make the room cooler”) (for additional examples, see the Appendix).

The participants received the following instruction in Japanese: “Is the following sentence acceptable in Japanese? All sentences are grammatically correct, so please judge on the basis of whether the combination of words within the brackets (【 】) sounds natural or unnatural.”

### 3.4 Data analysis

A linear mixed-effects model (LMEM) (Baayen et al., 2008) was developed, in which collocation type, LOR and their interaction served as the fixed factors and participants and items were included as random factors. The three collocation types were sum coded. The variable LOR was treated as a categorical variable and coded on the basis of a simple coding system, resulting in two comparisons: NSs vs. long-LOR learners and NSs vs. medium-LOR learners. The random effects included the intercepts of the participants and the items^6^.

## 4 Results

**Figure 4:**
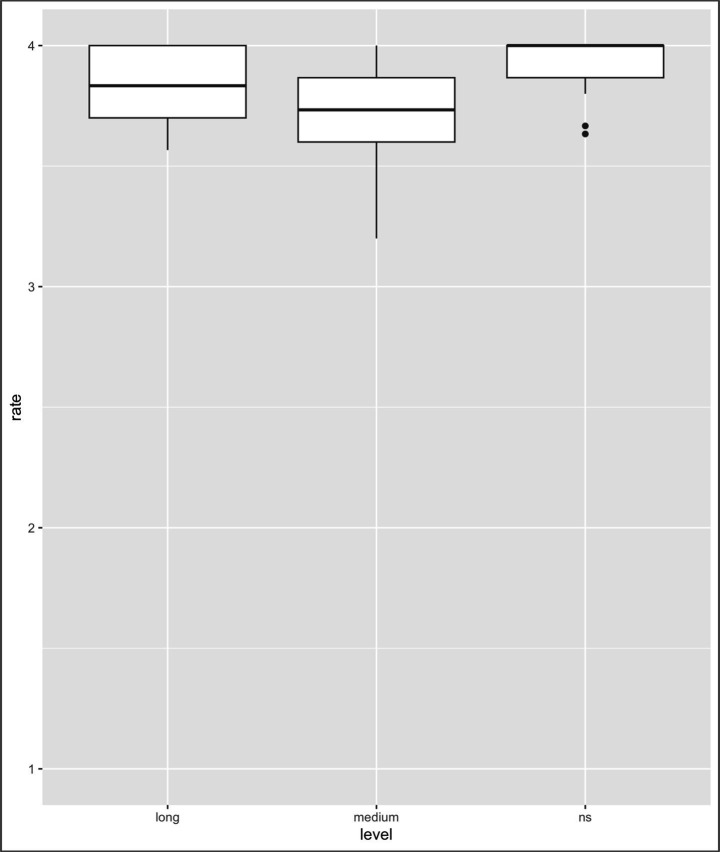
Results of the analysis of L1-L2 collocations. Mean rating of lexical judgment (1 = not acceptable, 2 = slightly unacceptable, 3 = slightly acceptable, 4 = acceptable) (note: Error bars represent standard errors).

**Figure 5:**
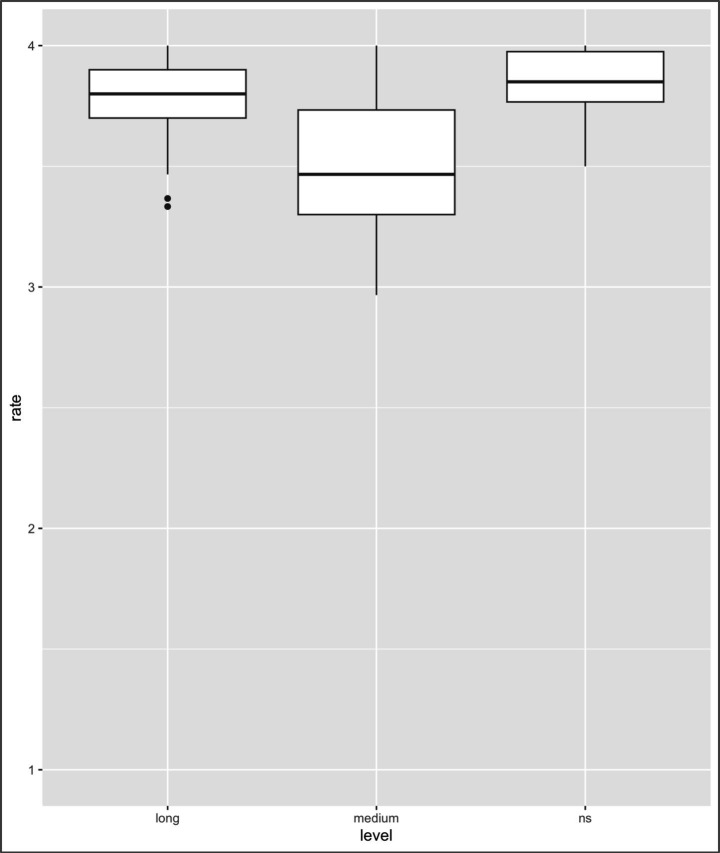
Results of the analysis of L2-only collocations. Mean rating of lexical judgment (1 = not acceptable, 2 = slightly unacceptable, 3 = slightly acceptable, 4 = acceptable) (note: Error bars represent standard errors).

**Figure 6:**
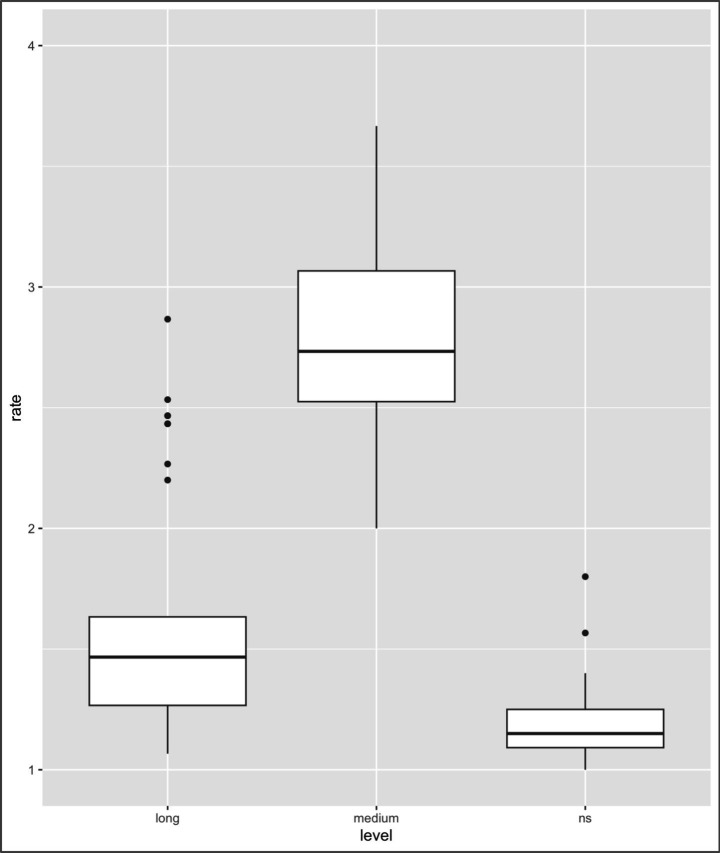
Results of the analysis of L1-only collocations. Mean rating of lexical judgment (1 = not acceptable, 2 = slightly unacceptable, 3 = slightly acceptable, 4 = acceptable) (error bars represent standard errors).

Table 3 summarizes the descriptive statistics (means, 95% confidence intervals, and Ns) of the acceptability ratings for each collocation type and group. With respect to the collocation type, the contrasts between L1–L2 vs. L1 only and L1–L2 vs. L2 only were significant (L1–L2 vs. L1 only: *β* = −2.05, SE = 0.01, *z* = −108.45, *p < 0*.001; L1–L2 vs. L2 only: *β* = −0.07, SE = 0.01, *z* = −3.80, *p* < 0.001), thus indicating that the ratings differed between these two types of collocations (i.e., L1–L2 collocations vs. L1 only and L2 only collocations). With regard to the LOR factor (see Figures 4–6), the contrasts between NSs vs. long-LOR learners and between NSs vs. medium-LOR learners were significant (NSs vs. long-LOR: *β* = −0.02, SE = 0.03, *z* = −0.82, *p* = 0.407; NSs vs. medium-LOR: *β* = −0.14, SE = 0.03, *z* = −4.83, *p* < 0.001). These results indicate that acceptability judgments varied systematically across collocation types and that the interpretation of higher or lower ratings is not uniform across conditions. Specifically, whereas higher ratings reflect greater accuracy for L1–L2 and L2-only collocations (see Figures 4, 5), lower ratings indicate greater accuracy for L1-only collocations (see Figure 5). Accordingly, the effect of collocation type was not uniform across conditions. L1–L2 collocations consistently received higher acceptability ratings than did L1-only collocations, reflecting shared and stable L1–L2 mappings. At the same time, L1–L2 collocations also differed from L2-only collocations, indicating that collocations patterns were not identically shared either with L2-only or with L1-only collocations. This asymmetry highlights qualitative differences in how collocation types are represented and evaluated by learners. Second, significant differences in ratings were observed between NSs and learners at different levels of LOR.

**Table 3:** Descriptive statistics of acceptability ratings by collocation type and group.

Collocation type	Group	*M*	95% CI	*N*
L1–L2	Long-LOR	3.08	[3.04, 3.13]	2,970
	Medium-LOR	3.33	[3.30, 3.36]	2,970
	NSs	2.99	[2.94, 3.04]	2,880
L2-only	Long-LOR	3.80	[3.77, 3.83]	990
	Medium-LOR	3.52	[3.48, 3.57]	990
	NSs	3.84	[3.81, 3.87]	960
L1-only	Long-LOR	1.62	[1.57, 1.68]	990
	Medium-LOR	2.76	[2.70, 2.83]	990
	NSs	1.19	[1.15, 1.22]	960

Additionally, three interactions associated with collocation type and LOR were observed, i.e., interactions with the contrast of L1-only and NSs vs. long-LOR learners (*β* = 0.70, SE = 0.04, *t* = 15.93, *p* < 0.001); L1-only and NSs vs. medium-LOR learners (*β* = 1.70, SE = 0.04, *t* = 39.04, *p* < 0.001); and L2-only and NSs vs. medium-LOR learners (*β* = −0.14, SE = 0.04, *t* = −3.27, *p* = 0.001); however, no interactions with the contrast of L2-only and NSs vs. long-LOR learners were observed (*β* = −0.04, SE = 0.04, *t* = −1.04, *p* = 0.297). The results are summarized in Table 4 and Figures 4–6.

**Table 4:** Analysis results.

Fixed effect	Estimate	Std. error	*t*-value	Pr(>|*t*|)
Intercept	3.8010	0.0134	283.553	<0.001
L1–L2 vs. L1 only	−2.0560	0.0189	−108.453	<0.001
L1–L2 vs. L2 only	−0.0722	0.0189	−3.809	0.001
NSs vs. Long	−0.0259	0.0313	−0.828	0.407
NSs vs. Medium	−0.148	0.0307	−4.838	<0.001
L1–L2 vs. L1 only * (NSs vs. Long)	0.705	0.0442	15.930	<0.001
L1–L2 vs. L2 only * (NSs vs. Long)	−0.046	0.0442	−1.042	0.297
L1–L2 vs. L1 only * (NSs vs. Medium)	1.700	0.0435	39.045	<0.001
L1–L2 vs. L2 only * (NSs vs. Medium)	−0.142	0.0435	−3.278	<0.001
Simple effects: rating of collocation type for each length of residence				
L1–L2: NS vs. Long	−0.025	0.083	−0.310	0.757
L1–L2: NS vs. Medium	−0.148	0.082	−1.812	0.073
L2: NS vs. Long	−0.072	0.050	−1.432	0.156
L2: NS vs. Medium	−0.291	0.049	−5.895	<0.001
L1: NS vs. Long	0.679	0.142	4.771	<0.001
L1: NS vs. Medium	1.551	0.140	11.075	<0.001

To obtain a clearer understanding of these interactions, we performed separate LMEMs for each of the collocation types, in which levels were included as the primary predictor of interest, with the aim of assessing the corresponding simple effect within each specific collocation type.

As Table 4 indicates, first, with respect to L1-L2 collocation, no significant differences were observed between long-LOR learners and NSs (*β* = −0.02, SE = 0.08, *t* = −0.31, *p* = 0.757) or between medium-LOR learners and NSs (*β* = −0.14, SE = 0.08, *t* = −1.81, *p* = 0.073); second, for L2-only collocation, significant differences were observed between NSs and medium-LOR learners (*β* = −0.07, SE = 0.05, *t* = −1.43, *p* = 0.156) but not between NSs and long-LOR learners (*β* = −0.29, SE = 0.04, *t* = −5.89, *p* < 0.001); third, with regard to L1-only collocation, significant differences were observed between NS and medium-LOR learners (*β* = 0.67, SE = 0.14, *t* = 4.77, *p* < 0.001) and between long-LOR learners and NSs (*β* = 1.55, SE = 0.14, *t* = 11.07, *p* < 0.001)^7^.

The results indicate that both long-LOR and medium-LOR learners rated L1-only collocations in a different manner than did NSs. Specifically, NSs rated L1-only collocations, which are anomalous in Japanese, significantly lower (i.e., more accurately) than did either medium-LOR or long-LOR learners. These findings suggest that although NSs reliably rejected L1-only collocations as anomalous, both long-LOR and medium-LOR learners exhibited a significantly weaker tendency to do so, thus indicating the persistent influence of L1 on their acceptability judgments.

The results presented above suggest the following. First, both medium- and long-LOR learners differ from NSs in terms of their collocation knowledge. Second, both medium-LOR learners and long-LOR learners rate L1-only collocations differently than do NSs. Third, medium-LOR learners find it difficult to rate L2-only collocations in a manner that is comparable to NSs; however, long-LOR learners do not; i.e., they behaved like NSs (Table 5).

**Table 5:** Summary of the results.

Collocation type	Significant difference
L1–L2 (correct)	NS = Long-LOR = Medium-LOR
L2-only (correct)	NS = Long-LOR > Medium-LOR
L1-only (incorrect)	NS > Long-LOR = Medium-LOR

## 5 Discussion

The present study examined how medium-LOR learners, who have lived in Japan for 3–7 years, and long-LOR learners, who have lived in Japan for more than 10 years, develop knowledge of L2 word meanings. In particular, this research focuses on native Chinese speakers learning Japanese and examines their acceptability judgments of six Japanese verbs: *akeru* (‘open’), *shimeru* (close), *sumu* (‘live’), *taberu* (‘eat’), *nomu* (‘drink’), *tsukuru* (‘make’), and *agaru* (‘go up’). These verbs have semantic domains in Japanese that differ from their Chinese counterparts.

The results of the acceptability judgment test revealed that medium-LOR learners, although performing in the same way as other groups in L1–L2 collocations, rated both L2-only collocations (which are correct in Japanese but not in Chinese) and L1-only collocations (which are incorrect in Japanese but correct in Chinese) in a different manner than did NSs. In contrast, long-LOR learners reported ratings different from NSs only for L1-only collocations, which suggests that neither medium-LOR nor long-LOR learners fully achieve a native-like knowledge of the relevant semantic domains. This finding supports the hypothesis developed by Jiang (2000), who proposed that L2 word learning stagnates during the second lemma mediation stage as a result of fossilization from L1 interference. Interestingly, unlike the foreign language learners investigated by Wolter and Yamashita (2018), the long-LOR learners included in our study appeared to be capable of processing (i.e., judging) L2-only collocations in a near-native manner before they could assess L1-only collocations accurately when they encountered L2 meanings. Whereas Wolter and Yamashita employed a primed lexical decision task to examine online collocational processing, the present study used an acceptability judgment task that required explicit evaluative judgments. Despite these task differences, both studies point to greater difficulty with L1-only collocations, suggesting a persistent influence of L1 semantic representations. This finding suggests that the task of acquiring L1-only collocations is more challenging than that of acquiring L2-only or L1–L2 collocations.

In the following sections, we discuss the developmental changes in L2 lexical-semantic acquisition between medium-LOR and long-LOR learners, the differences in ratings among the various collocations, and the overall attainment of L2 semantic knowledge in this context.

### 5.1 Developmental changes in lexical-semantic knowledge by medium-LOR vs. long-LOR L2 learners

The most salient pattern observed in the present study is that long-LOR learners showed clear improvement over medium-LOR learners in their acceptability judgments of L2-only collocations yet continued to diverge from native speakers in terms of their evaluation of L1-only collocations. This pattern suggests that extended exposure facilitates partial lexical-semantic development, while L1-mediated representations remain resistant to full restructuring even after long-term residence over 10 years.

Jiang (2000) argued that while L2 word learning can theoretically progress through several stages—i.e., formal, lemma mediation, and beyond—it often stalls at the lemma mediation stage as a result of the fossilization associated with the influence of L1. As Jiang noted, “given the presence of the L1 lemma in the lexical entry, contextualized exposure may also automatically reinforce L1 lemma mediation by strengthening the connection between the L1 lemma and the L2 lexeme” (Jiang, 2000, pp. 54–55). L2 learners frequently access word concepts via their L1 when they encounter L2 words. Consequently, the transition from L1 lemma mediation to L2 integration may take longer than expected, as learners rely on their L1 for meaning, thus reducing their sensitivity to L2-specific constraints (Jiang, 2000). This developmental asymmetry can be interpreted directly within Jiang’s (2000) lexical development model, and our results support this theoretical account. In the rating of L1-only (i.e., incorrect) collocations, both medium-LOR and long-LOR learners accepted more of these anomalous combinations than did NSs, thus indicating the persistent influence of L1. This result is consistent with the findings reported by Long (2003), who documented fossilized lexical errors in English in a Japanese speaker, Ayako, after more than 40 years of residence in an L2-speaking environment. Similarly, Jiang (1999) demonstrated that even proficient L2 English learners who have resided in the United States for extended periods had difficulty with certain forms of lexical priming, thus suggesting that their L2 lexical systems remained partially reliant on L1 mediation.

On the basis of Jiang’s three-stage model, the medium-LOR learners in the present study are best characterized as remaining at the lemma mediation stage (second stage), as evidenced by their difficulty with both L1-only and L2-only collocations. In contrast, the long-LOR learners showed signs of progression toward the integration stage (third stage), particularly in their judgments of L2-only collocations, where their performance was comparable to that of NSs. However, their continued overacceptance of L1-only collocations indicates that this progression remains incomplete and that L1-based representations have not been fully suppressed even after more than 10 years of residence in Japan.

Building on Jiang’s model, subsequent research has extended its scope from single lexical items to collocational learning. Yamashita and Jiang (2010), for instance, investigated learners’ processing of congruent and incongruent collocations and found that learners had greater difficulty with incongruent items, as evidenced by slower reaction times. However, their analysis did not distinguish between two types of incongruence—collocations that are acceptable only in the L2 but not in learners’ L1 (L2-only), and those that are acceptable in the L1 but not in L2 (L1-only).

The present findings demonstrate that these two types of incongruences follow distinct developmental trajectories. Indeed, our findings suggest that L1-only collocations—those that are acceptable in L1 (Chinese) but anomalous in L2 (Japanese)—pose particular challenges even for long-LOR learners, thus highlighting the persistence of L1-induced processing strategies. In contrast, L2-only collocations appear to be amenable to change with extended exposure, reflecting developmental progress toward lexical integration. Taken together, these results indicate that extended residence promotes movement toward the L2 integration stage but does not guarantee full semantic restructuring when L1-based collocational patterns conflict with L2 norms. Differences between L1-only and L2-only collocations are discussed in further detail in Section 5.3.

### 5.2 Input effects on collocation learning

The current study revealed that both medium-LOR and long-LOR learners can correctly judge L2-only collocations more easily than they can L1-only collocations. Similar results have been reported by other studies that have involved different methodologies and languages (Wolter and Yamashita, 2015, 2018). For example, Wolter and Yamashita (2018) focused on an acceptability judgment task in which L1 Japanese learners of English were compared with native English speakers. These authors revealed that learners’ reaction times in the context of judging L1-only collocations were slower than those that they exhibited for L2-only collocations and for L1–L2 congruent collocations. While these authors acknowledged the importance of explaining why L1-only collocations do not offer a processing advantage, they did not provide a specific explanation for this observation. To address this gap, we examine the influence of input effects in relation to positive and negative evidence.

Unsurprisingly, L2-only collocations are acquired through consistent exposure to such exemplars in the form of input in L2-speaking environments, thus making this type of collocation more prevalent in the daily input of L2 learners than are L1-only collocations, which are practically absent in the input. Previous studies have generally identified the frequency of occurrence as one of the best predictors of collocation usefulness and acquisition (Durrant and Schmitt, 2010; Fernández and Schmitt, 2015). On the basis of a collocation test administered to L2 learners, Durrant and Schmitt (2010) suggested that advanced nonnative English speakers use collocations that frequently occur in their input. Similarly, González Fernández and Schmitt (2015) identified a correlation between L2 learners’ test scores in collocational knowledge of L2 Spanish and the corpus frequency of collocations, thus indicating that exposure frequency significantly influences acquisition. Furthermore, Wolter and Gyllstad (2013) highlighted the importance of entrenchment, in which context frequent input promotes the activation of certain language structures, thus transforming specific words or collocations into more automated routines (Schmid, 2010). In essence, more frequent structures and collocations are more likely to become entrenched than are less frequent examples.

In the present study, we observed clear input effects on judgments concerning L2-only and L1-only collocations. The former type often appears in learners’ daily input, whereas L1-only collocations do not appear in L2 input. Most of the L2-only collocations that were included in this study were highly frequent in the target language (Japanese), such as *denki-o tsukeru* (‘turn on the light’), *hoteru-ni tomaru* (‘stay in a hotel’), and *terebi-o kesu* (‘turn off the television’), each of which occurred at least 10 times in a native Japanese corpus (*M* = 621 times, as noted earlier). The relatively high frequency of these L2-only collocations likely facilitated their acquisition in both medium-LOR and long-LOR learners, contributing to their more target-like acceptability judgments.

The significance of input frequency is consistent with the usage-based model of language (Bybee and Hopper, 2001), which posits that language acquisition is an experiential process that is highly sensitive to frequency effects. This perspective has been proposed in the context of child L1 acquisition (e.g., Kidd et al., 2010; Lieven, 2014; Tomasello, 2003), suggesting that children closely imitate the language that they hear. It has also been used to explain L2 development in adult learners (Wolter and Gyllstad, 2013; Ellis and Wulff, 2020). From this perspective, frequent exposure provides abundant positive evidence, thereby strengthening L2-specific form–meaning mappings and promoting lexical-semantic integration. However, it should be noted that item frequency may also have influenced the collocation acceptance patterns observed in Figure 4 (L1–L2 collocations) and 5 (L2-only collocations). Highly frequent collocations may be readily accepted even by long-term residents, not necessarily as a result of length of residence per se but rather due to the cumulative effect of repeated encounters over time. While the present study focused on differences associated with length of residence, it is important to recognize that length of residence constitutes a composite measure that inherently encompasses language exposure and usage opportunities. Accordingly, future research should consider item frequency and cumulative exposure as integral components of length-of-residence effects rather than treating them as separable influences.

Importantly, however, frequency alone cannot account for the full pattern of results observed in the present study. If acceptability judgments were driven primarily by input frequency, then L1-only collocations—whose occurrence in Japanese input is virtually nonexistent—should have received uniformly low ratings from L2 learners. Contrary to this expectation, both medium-LOR (M = 2.76) and long-LOR (M = 1.62) learners consistently rated L1-only collocations as relatively acceptable (see Table 3, bottom). This pattern suggests that learners’ judgments are not solely determined by frequency-based entrenchment in the L2 but instead reflect persistent L1-mediated lexical-semantic representations, consistent with Jiang’s (2000) lemma mediation stage, in which L2 forms continue to access meaning via entrenched L1 lexical representations.

Taken together, these findings suggest that while input frequency plays a crucial role in the acquisition and entrenchment of L2-only collocations, it does not fully explain learners’ acceptance of L1-only collocations. Rather, the present results underscore the interaction between input effects and cross-linguistic influence, highlighting the importance of considering both positive evidence from L2 input and the absence of reliable negative evidence against L1-based patterns (to be discussed in the next section).

### 5.3 Positive and negative evidence in the context of reconstructing L2 semantic knowledge

The difficulties that L2 learners face in the process of judging L1-only collocations in comparison with L2-only collocations may be the result of the availability of positive and negative evidence in their input. It is generally more difficult for L2 learners to produce correct judgments concerning L1-only collocations, which require the learner to infer the unacceptability of such collocations on the basis of negative evidence—often in the form of corrective feedback—than to evaluate L2-only collocations, which can be acquired through repeated exposure to correct forms (i.e., positive evidence). This pattern is consistent with previous research that has reported that L2 learners tend to struggle more with structures that are permissible in L1 but anomalous in L2, whereas they encounter fewer difficulties with L2-specific structures that lack equivalents in L1 (Inagaki, 2001; Nishi and Shirai, 2021), thereby illustrating the asymmetrical roles played by positive and negative evidence in L2 development. Importantly, pedagogical and psycholinguistic studies have shown that such difficulties can be alleviated when learners receive explicit negative evidence or corrective feedback. For example, research on form-focused instruction suggests that drawing learners’ attention to L2–L1 mismatches and explicitly indicating anomalous combinations can facilitate restructuring in domains where positive evidence alone is insufficient (e.g., White, 1987). These findings suggest that the persistent acceptance of L1-only collocations may partly reflect the absence of systematic negative evidence in naturalistic input.

Inagaki (2001) examined the acquisition of motion verbs (such as *walk* and *run*) used with goal prepositional phrases (PPs) contained in sentences such as “*John-ga gakkoo-ni aruita” ‘John walked to school’, which is unacceptable in Japanese. English, the L1 of the participants in this research, allows a wider variety of motion verbs to be paired with goal PPs than does Japanese, which permits only directed-motion verbs in such contexts. Inagaki concluded that “L1 influence persists when an argument structure in the L2 constitutes a subset of its counterpart in the L1” (Inagaki, 2001, p. 153).

A more recent study conducted by Nishi and Shirai (2021) revealed similar trends in the ways in which native English, Chinese, and Korean speakers learned a Japanese aspect marker *-teiru*. Learners found the task of rejecting aspectual structures that are impossible in L2 (e.g., *Satoo-san wa tookyoo ni sumimasu, which is intended to mean ‘Mr. Sato lives in Tokyo’) to be more difficult than that of accepting grammatical structures that are possible in L2 (e.g., *Satoo-san wa sofa ni suwatte imasu* – ‘Mr. Sato is sitting in the sofa’).

In line with previous research on the impacts of positive and negative evidence, the current study revealed that learners struggle to reject collocations that are acceptable in their L1 but not in their L2, likely as a result of a lack of negative evidence to indicate the impossibility of L1-only collocations, such as *hoteru ni sumu ‘live in a hotel’.

How can we explain the discrepancy between the rejection of incorrect sentences and the acceptance of grammatical sentences? We suggest that this divergence arises from the limitations of implicit learning in adult L2 learners (Shirai, 2019, pp. 21–26). Ellis (2004, 2005, 2006) explored the roles played by explicit and implicit knowledge in various L2 tasks and reported that implicit knowledge is correlated with the ability to judge grammatical sentences correctly, whereas explicit knowledge is related to the rejection of anomalous sentences. In contrast, in the process of L1 acquisition, implicit learning on the basis of input processing enables children to judge both grammatical and ungrammatical sentences correctly, thus indicated that positive evidence alone as sufficient for learners to achieve native-level competence (Goldberg, 2003). For L2 learners, however, implicit learning on the basis of positive evidence is often inadequate. At advanced levels, learners may at least accept correct sentences on the basis of their exposure to positive evidence, which indicates that a structure is possible in L2. Our study reveals that for such items, L2 learners with over 10 years of residence exhibit similar performance to that of NSs.

In contrast, exposure to positive evidence does not inform learners about ungrammatical sentences. The task of rejecting an ungrammatical sentence is challenging, as learners typically do not receive feedback indicating that a sentence is incorrect when, for instance, they say *denki-o akeru (‘open the light’). This difficulty is exacerbated when the translation equivalent is grammatical in the learner’s L1 (as found for L1-only collocations in our study). Consequently, the strong influence of L1 translation remains even after prolonged exposure to the target language. Lexico-semantic transfer is pervasive and persistent (Tanaka and Abe, 1985) even after 10 years of language experience in input-rich environments. To mitigate such negative transfer, the consistent application of negative evidence that involves explicit knowledge (e.g., noting that one cannot say *denki-o akeru) may be necessary. This negative evidence is often absent in naturalistic L2 learning contexts unless the learner is also enrolled in an L2 classroom.

### 5.4 Ultimate attainment in semantic acquisition

Research has indicated that a residency of more than 10 years in an L2-speaking country is generally sufficient to enable learners to achieve a relatively stable level of proficiency in L2. During this stage, learners can often be viewed as near-native in certain linguistic domains (Bialystok and Hakuta, 1999; Birdsong and Flege, 2001; Lardiere, 2007; Shirahata, 2006), particularly in terms of their syntactic ability (Lardiere, 2007; Shirahata, 2006). However, this level of proficiency is not always obtained. Some researchers, such as Abrahamsson and Hyltenstam (2009), have reported that late L2 learners—i.e., those who begin learning the language after adolescence—often struggle to attain the same performance as NSs on various tests, including grammar, pronunciation, and vocabulary tests. With respect to semantic ability—particularly phrasal semantics—Slabakova (2006) reviewed neurophysiological studies and argued that L2 learners can, in some cases, develop native-like processing patterns. For example, studies such as those conducted by Weber-Fox and Neville (1996) and Hahne (2001) have provided evidence indicating that advanced L2 learners are capable of processing phrasal-level semantic anomalies in ways that are comparable to the performance of NSs.

Notably, a study conducted by Jiang (1999), which focused on the semantic knowledge of advanced L2 learners, revealed that the representational status of the L2 lexical system remained unchanged, even among highly proficient learners. However, importantly, the maximum LOR for participants in Jiang’s study was 7 years, which is shorter than the 10 years that have been considered in other studies on ultimate attainment (e.g., Bialystok and Hakuta, 1999; Shirahata, 2006).

In the present study, we observed that even learners who had more than 10 years of residency in an L2-speaking country and who had reached the highest level on the Japanese Language Proficiency Test (JLPT) still exhibited differences from NSs in terms of their judgment of L2 collocations. This finding may suggest that the goal of achieving native-like L2 semantic knowledge may not be feasible for most learners. At first glance, our findings may suggest limits on the attainment of native-like L2 semantic knowledge. However, it should be noted that the present study adopted a cross-sectional design, which does not allow for definitive conclusions about developmental end states to be drawn. Our study examined the influence of LOR in the target language environment on the basis of a comparison between long-term and medium-term residents. To ensure that the participants in this research were advanced learners, we specifically selected individuals with JLPT Level N1 certification, which has been reported to correspond to levels of proficiency that are comparable to CEFR C1—that is, the ability to comprehend and produce complex academic and professional texts with a high degree of fluency and accuracy (Hamada, 2019; Nishizawa et al., 2022).

Our results show while L2 learners with extended exposure may approximate native-like performance in some areas, such convergence is selective and contingent on the type of collocational relationship between the L1 and L2 rather than uniform across all semantic domains. Despite their long residence in an L2-speaking environment, significant differences were observed between NSs and both long-LOR and medium-LOR learners in terms of their judgments concerning certain collocation types, particularly L1-only collocations. In contrast, no significant differences from NSs were observed for long-LOR learners with respect to L1–L2 collocations and for long-LOR learners with respect to L2-only collocations.

More broadly, these findings support Lardiere’s (2007) conclusion that L2 end states can be native-like in certain respects but not in others. On the basis of an acceptability judgment test, we observed that L2 learners can achieve native-like semantic knowledge through exposure to frequent input—abundant positive evidence—especially when L1–L2 mapping is congruent or when the L2 form is acceptable. At the same time, the present results show that extended exposure alone may not be sufficient for fully restructuring lexical-semantic representations when L1-based patterns remain strongly entrenched, particularly in the absence of clear negative evidence. Lexical-semantic transfer is pervasive and persistent, and long-term residence may not be an answer to overcoming such negative transfer.

## 6 Conclusion

This study examined whether adult L2 learners with more than 10 years of exposure to Japanese in a target language environment can attain native-like performance in the context of judging the acceptability of lexical items that may differ in collocational restriction, in contrast to L2 learners with 3–7 years of exposure. The results of this research indicate that even long-term residents of 10+ years struggle to acquire lexical-semantic knowledge equivalent to that of NSs, especially when a given meaning is acceptable in L1 but anomalous in L2. The learners who participated in this research often failed to identify such expressions as unacceptable. In contrast, L2-only meanings—i.e., those that are correct in Japanese but absent in L1—were more easily identified as acceptable, and extended exposure (above 10 years) led to judgments that were equivalent to the norms observed among NSs, while 3–7 years of residence is not enough for this achievement.

These findings support the claim that L2 lexical acquisition progresses through distinct stages—i.e., the formal, lemma mediation, and integration stages (Jiang, 2000)—but that development may plateau at the lemma mediation stage, especially when L1-based processing persists. Importantly, however, the present findings should not be taken to suggest that ultimate attainment in L2 semantic knowledge is categorically impossible. Rather, the cross-sectional design of the study limits our ability to determine whether the observed differences reflect stable end states or delayed but ongoing semantic restructuring.

The study highlights the enduring influence of L1 on L2 lexical-semantic development, particularly in the case of collocations that are acceptable in L1 but anomalous in L2. While behavioral judgment tasks reveal persistent differences between learners and native speakers, they may not fully capture gradual changes at the neural level. This research highlights several directions for future research. First, further investigation is needed to clarify the cognitive and input-related factors that can promote or inhibit progression from lemma mediation to integration. While LOR contributes to development, our findings suggest that exposure alone may be insufficient to overcome deeply entrenched L1-based patterns. Future research should explore the roles played by input quality, output opportunities, metalinguistic awareness, particularly via negative evidence, and age of acquisition in this context. Second, while this study focused on verb–noun collocations, future researchers should investigate whether similar patterns emerge in the context of other multiword expressions (e.g., idioms, prepositional phrases, or light verb constructions) with the goal of determining whether the observed difficulties are specific to collocational knowledge or generalizable to other lexical-semantic domains.

Finally, future research adopting longitudinal and neurolinguistic approaches might help clarify whether advanced L2 learners can eventually restructure their semantic representations given sufficient and appropriate input. For example, event-related potential (ERP) studies could examine whether advanced learners exhibit increasingly native-like N400 responses to collocational violations over time, which would indicate deeper semantic integration. Longitudinal designs combining behavioral and neurophysiological measures would be particularly informative in determining whether the differences observed in the present study reflect stable end states or delayed, but ongoing, semantic development.

## Data Availability

The datasets presented in this study can be found in online repositories. The names of the repository/repositories and accession number(s) can be found in the article/Supplementary material.

## Footnotes

1 The preposition *am* in this context suggests that a specific time or date should follow, but such a reference is missing in this sentence. In German, *am* typically refers to a specific day or date, like *am Samstag* ‘on Sunday’ (*Der Geschäft wurde am Samstag geschlossen*: The store was being on Saturday closed). Without this point, the sentence feels incomplete; thus, this sentence contains a syntactic violation.

2 O is an accusative case marker in Japanese.

3
https://j-test.jp/cefr-jtest

4
https://tsukubawebcorpus.jp/en/

5 The four-point scale was selected to balance interpretability and reliability in an L2 population (Schütze and Sprouse, 2013). Finer-grained scales were not adopted because they require subtle distinctions that may not be made consistently by L2 learners.

6 Analyses were conducted using a linear mixed effects model with collocation type and length of residence as fixed effects and random intercepts for participants and items *(lmer(rate ~ CollocationType * LengthResidence + (1 | subj) + (1 | item))).*

7 As a supplementary analysis, we examined whether item frequency was associated with item-level accuracy for L1–L2 and L2-only collocations among long-LOR learners. Pearson correlation analyses revealed no significant relationship for L1–L2 collocations (*r* = 0.04, *p* = 0.84) and a weak, non-significant, negative correlation for L2-only collocations (*r* = −0.24, *p* = 0.20), meaning low-frequency items tend to be given lower ratings for acceptability. While these analyses were exploratory and limited in scope, they suggest that item frequency may play some role for L2-only items but not for L1–L2 items. This is understandable because for L2-only items, it would be easier for learners to rely on positive evidence encountered in L2 input in Japanese, while in L1–L2, it would be easier for learners to rely not only on positive evidence but also on L1 equivalents in Chinese, which positively constrain lexical development (i.e. positive transfer from L1 to L2). This question should be addressed in future research.
