# Supplementary material for: Ultimate attainment in L2 semantics: Where and why do learners fail to achieve native-like knowledge of verb meaning?
Source: Front Psychol. 2026 Apr 16;17:1719015. doi: 10.3389/fpsyg.2026.1719015 (PMC13128554; doi:10.3389/fpsyg.2026.1719015)
Supplement: Supplementary file 1 [file Data_Sheet_1.pdf]

Appendix 1. Examples of test sentences for L2-only collocations

L2-only				
1, *Kurumaisu no hito ga tooruk kara michi o* 【*akete*】 *kudasai*<br>[michi **akeru**: open road]<br>(Please open the road because a person in a wheelchair is passing)	1 unacceptable	2 slightly-unacceptable	3 slightly-acceptable	4 acceptable
2, *Mogura ga jimen ni ana o* 【*akete*】 *imasu*<br>[ana **akeru**: open hole]<br>(The mole rat is opening a hole in the ground)	1	2	3	4
3, *Tantousha ga seki o* 【*aketeiru*】 *node nochihodo mata orikaeshimasu*<br>[seki **akeru**: open seat]<br>(The person in charge is away (open) from their seat so we will call you back later)	1	2	3	4
4, Mae no hito to kannkaku o 【*akete*】 *narande kudasai*<br>[kankaku **akeru**: open space]<br>(Please line up while opening space from the person in front)	1	2	3	4
5, *Mae no hito tono sukima o* 【*akenaide*】 *narande kudasai*<br>[sukima **akeru**: wear the boots]<br>(Please line up without opening space between the person in front)	1	2	3	4
6, *Kono shorui no sakusei niwa ichigyou no gyoukan o* 【*akete*】 *kudasai*<br>[gyoukan **akeru**: open line]<br>(While creating this document, please open one line)	1	2	3	4
7, *Yakei ga kirei nanode yoru demo hotondo kaaten o* 【*shimemasen*】<br>[kaaten **shimeru**: close curtain]<br>(The scenery at night is beautiful so I don't close the curtain often)	1	2	3	4
8, *petto botoru no futa o* 【*shimenai*】 *to taoreta toki ni nakami ga dete shimaimasu*<br>[futa **shimeru**: close bottle]	1	2	3	4

(If you don't close the lid on the plastic bottle, the contents will come out when it falls over)				
9, *Ie o deru toki wa kanarazu kagi o【shimete】 kudasai*<br>[kagi **shimeru**: close key]<br>(Please lock (close) the door when exiting the house)	1	2	3	4
10, *Mabushii node buraindo o 【shimemasu】*<br>[buraindo **shimeru**: close blind]<br>(It is bright so I will close the blinds)	1	2	3	4
11, *Samukattara jyaketto no jyanpaa o【shimetara】?*<br>[jyanpaa **shimeru**: close zipper]<br>(If you are cold, why don't you close the jacket zipper?)	1	2	3	4
12, *Guai ga warui node okayu o 【tabete】 yukkuri yasumi masu*<br>[okayu **taberu**: eat porridge]<br>(I don't feel well so I will eat porridge and take a rest)	1	2	3	4
13, *Chuukagai de shouronpou o 【tabeta】 ato harusame suupu mo tabetayo*<br>[suupu **taberu**: eat soup]<br>(After eating soup dumping, I ate glass noodle soup too in China Town)	1	2	3	4
14, *Maiasa nyuusankin iri yooguruto o 【tabete】imasu*<br>[yooguruto **taberu**: eat yogurt]<br>(Every morning, I eat yogurt with lactic acid bacteria in it)	1	2	3	4
15, *Kusuri o mikka mo nomiwasurete 【shimat】te doushiyou*<br>[kusuri **nomu**: drink medicine]<br>(I forgot to take (drink) medicine for three days. What should I do?)	1	2	3	4
16, *Kaze o hiite shimai tsuba o 【nomu】 to nodo ga totemo itai desu*<br>[tsuba **nomu**: drink saliva]<br>(Because I got sick, my throat hurts when swallowing (drink) my saliva)	1	2	3	4

17, *Kore izhou sensou o tsuzukeru wake niwa ikanai node teki no kuni no zhouken o* 【*nonda*】<br>[zhouken **nomu**: drink condition]<br>(We don't want to continue the war anymore so we followed (drink) the condition of the enemy country)	1	2	3	4
18, *Kodomo niwa yoi omoide o takusan* 【*tsutte*】 *ageyou*<br>[omoide **tsukuru**: make memory]<br>(Let's create (make) lots of good memories for the children)	1	2	3	4
19, *Kodomo ga suki nanode shourai wa kodomo sannin kurai* 【*tsukuritaina*】<br>[kodomo **tsukuru**: make children]<br>(I like kids, so I want to have (make) three kids in the future)	1	2	3	4
20, *Supoutsu ni tsuyoi karada o* 【*tsukuru*】 *zo*<br>[karada **tsukuru**: make body]<br>(I will make a strong body for sports!)	1	2	3	4
21, *Ame ga* 【*agatta*】 *younano de shiai saikai dekisou da*<br>[ame **agaru**: rain go up]<br>(The rain stopped (go up) so we can restart the competition)	1	2	3	4
22, *Kono an wa nando mo giron ni* 【*aggatte*】 *inagara jitsugen saremasen deshita*<br>[giron **agaru**: topic go up]<br>(Even though the topic came up (go up) many times, it was not created)	1	2	3	4
23, *A to B ga wagasha no saiyuusyuu no kouho ni* 【*agatta*】<br>[kouho **agaru**: candidate go up]<br>(The top items A and B from our company were selected (go up) as candidates)	1	2	3	4
24, *Saikin chikyuuondanka ya taikiosen ga yoku wadaini* 【*agatte*】 *imasu*<br>[wadai **agaru**: topic go up]	1	2	3	4

(Recently, global warming and air pollution as been a rising (go up) trending topic)				
25, *Natsuyasumi no ryokou de goukana hoteru ni* 【*tomatte*】 *daimanzoku*<br>[hoteru **tomaru**: stay hotel]<br>(I am satisfied after staying (stay) at a luxurious hotel during my summer vacation)	1	2	3	4
26, *Kyanpu ni kita minasanha tugi no dono tento ni* 【*tomari*】*taidesuka*<br>[tento **tomaru**: stay tent]<br>(Everyone that came to the camp, which of these next tents would you like to stay (stay) in?)	1	2	3	4
27, *Konkai no onnsennryokou dewa kono chiisana yado ni* 【*tomari*】 *masu*<br>[yado **tomaru**: stay hut]<br>(During this hot spring vacation, I will stay (stay) at this small hut)	1	2	3	4
28, *Shuugakuryokou dewa nikkou ni aru shukusya ni* 【*tomaru*】 *koto ni natteiru*<br>[shukusya **tomaru**: stay hotel]<br>(During the field trip, we will stay (stay) in a hostel with Nikkou)	1	2	3	4
29, *Kazoku ryokou de furui ga fuzei aru ryokan ni* 【*tomatta*】<br>[hoteru **tomaru**: stay hotel]<br>(During our family trip we will stay (stay) in a Japanese style hotel that is old but elegant)	1	2	3	4
30, *Kyou wa mou osoinode kono yamagoya ni* 【*tomatte*】 *ashita gezan shiyou*<br>[yamagoya **tomaru**: stay hut]<br>(It is already late today so let's stay (stay) in this hut and go down the mountain tomorrow)	1	2	3	4

Appendix 2. Examples of test sentences for L1-only collocations

L1-only				
1, *Pasokon ga uirusu ni kansen shite shimau osore ga arukara meiwaku meeru de kita saito wa zettai ni* 【*akeruna*】<br>[saito **akeru**: open link]<br>(Do not open the link on the email because you might get virus form it.)	1 unacceptable	2 slightly-unacceptable	3 slightly-acceptable	4 acceptable
2, *Denkidai no muda nanode denki wo* 【*ake*】*ppanashi ni shinaide ne*<br>[denki **akeru**: open light]<br>(Keep the electricity open (turn on) since it is a waste of money)	1	2	3	4
3, *Mizu o takusan yatta node akai kireina hana ga* 【*aki*】*mashita*<br>[hana **aku**: open flower]<br>(I gave it a lot of water so a beautiful red flower opened (blossomed))	1	2	3	4
4, *Totemo atsui node eakon o* 【*akete*】 *heya no onndo o sagemasu*<br>[eakon **akeru**: open air conditioner]<br>(It is very hot so we will open (turn on) the air conditioner to make the room cooler)	1	2	3	4
5, *Terebizuki no kodomo wa gakkou kara kaettekuru to terebi o* 【*ake*】*youto suruga mazu wa shukudai o yaraseru youni shiteimasu*<br>[terebi **akeru**: open television]<br>(whenever children who like watching tv come back from school, they try to open (turn on) the tv but first I try to make them do homework)	1	2	3	4
6, *Kitaguni dewa sutoobu o* 【*akenai*】*to fuyuba wa samukute kaitekini sugosu koto ga dekimasen*<br>[sutoobu **akeru**: open stove]<br>(In norther Japan we need to open (turn on) the stove in order to spend winter comfortably)	1	2	3	4
7, *Daremo miteinai node terebi o* 【*shimete*】 *kudasai*	1	2	3	4

[terebi **shimeru**: close television]<br>(No one is looking so please close (turn off) the television)				
8, *Heya no denki o 【shimete】 neru jyunnbi o shimasu*<br>[denki **shimeru**: turn off light]<br>(We will close (turn off) the room light to get ready for bed)	1	2	3	4
9, *Nerutoki wa sutoobu o 【shimenai】 to kaji no kikensei ga atte abunai yo*<br>[sutoobu **shimeru**: close heater]<br>(we need to close (turn off) the heater when we sleep because it may cause a fire and its dangerous)	1	2	3	4
10, *Denki o 【shimete】 setsuyaku shimashou*<br>[denki **shimeru**: close light]<br>(Let's conserve the energy by closing (turning off) the air conditioner)	1	2	3	4
11, *Nerutoki wa senpuuki o 【shimenai】 to karada ni warui yo*<br>[senpuuki **shimeru**: close fan]<br>(When sleeping at night, it is bad for the body if the fan is not closed (turned off))	1	2	3	4
12, *Gohan no ato ni kono suiminyaku o 【tabete】 kudasai*<br>[suiminyaku **taberu**: eat sleeping pills]<br>(Please eat (take or drink) this sleeping pills after the meal)	1	2	3	4
13, *Seigo sankagetsu no akachan ga oishisou ni miruku o 【tabete】imasu*<br>[miruku **taberu**: eat milk]<br>(A three months old baby is deliciously eating (drinking) the milk)	1	2	3	4
14, *Kusuri o sannkai mo tabewasurete 【shimatta】 node isha ni akirerare mashita*<br>[kusuri **taberu**: eat medicine]<br>(The doctor was appalled (shocked) because I ate (take or drink) the medicine three times)	1	2	3	4

15, *Chuugoku nado no ajia no kuni dewa asagohan toshite chuukagayu o 【nomu】 hito ga ooi*<br>[chuukagayu **nomu**: drink rice gruel]<br>(In asian countries such as china, many people drink (eat) rice gruel for breakfast)	1	2	3	4
16, *Kaze o hiita node okayu o 【nonnde】 yasunnde imasu*<br>[okayu **nomu**: drink rice gruel]<br>(I got sick so I am drinking (eat) rice gruel and resting)	1	2	3	4
17, *Asa no dezaato toshite kudamono ga haitteiru yooguruto wo 【taberu】 noga nikka desu*<br>[yooguruto **nomu**: drink yogurt]<br>(My routine is drinking (eating) fruits in yogurt for dessert every morning)	1	2	3	4
18, *Kinou no yoru 【tsukutta】 yume nanndakke?*<br>[yume **tsukuru**: make dream<br>(What was the dream I made (saw) yesterday?)	1	2	3	4
19, *Kodomo wa ie ni kaettkuruto suguni shukudai o 【tsukuri】 hajime masu*<br>[shukudai **tsukuru**: make homework]<br>(When kids get home, they immediately make (do) their homework.)	1	2	3	4
20, *Shinseki wa daitokai de ookina shoubai o 【tsukutte】 ite totemo moukatte imasu*<br>[shoubai **tsukuru**: make buisness]<br>(My relatives are making (doing) business in a big city and are making lots of money)	1	2	3	4
21, *A! basu kita! Hora hayaku basu ni 【agatte】 hasshua shichuauyo*<br>[basu **agaru**: go up bus]<br>(Oh, the bus came! Hurry, go up (get on) the bus or the bus will leave)	1	2	3	4
22, *Zhuugo fun mae made ni geeto ni ikanaito hikouki ni 【agare】 naku nacchau yo*<br>[hikouki **agaru**: go up airplane]<br>(If we don't go to the gate by 15 minutes before, we cannot get up (get on) the airplane)	1	2	3	4

23, Koko chuusha kinshi dakara hayaku kuruma ni 【agatt】e<br>[kuruma **agaru**: car go up]<br>(Parking is not allowed here so hurry and get up (get in) the car)	1	2	3	4
24, *Hayaku densha ni 【agatte】 kore nogashitara tsugi wa sanjyuppungo nandakara*<br>[densha **agaru**: go up train]<br>(Hurry, get up (get on) the train! If we miss this one, the next one will be 30 minutest later)	1	2	3	4
25, *Kokugai shucchou de ikkagetsuhodo nyuyooku no koukyiuu hoteru ni 【sunda】 koto ga aru*<br>[hoteru **sumu**: live hotel]<br>(I stayed (lived) at a luxury hotel in newyork for a month for a business trip)	1	2	3	4
26, *Tozan ni kita minasan konnya wa kono tento ni 【sumimasu】 yo*<br>[tento **sumu**: live tent]<br>(Everyone that came to the camp, which of these next tents would you like to stay (stay) in?)	1	2	3	4
27, *Natsuyasumi wa sanshuukan hodo nouson no yado ni 【sund】e shizen ni shitashinde mitai to omou*<br>[yado **sumu**: rural house live]<br>(For summer vacation I want to live (stay) at a rural house and enjoy the nature)	1	2	3	4
28, *Shuugakuryokou dewa kyoto o kankou shita ato shukushua ni modotte seito mo sensei mo soko ni futaban 【sumi】masu*<br>[shukusha **sumu**: live accommodation]<br>(After sightseeing on a school trip in Kyoto, the students and teachers returned to their accommodation and lived (stayed) there for two nights)	1	2	3	4
29, *Yuujin to nihakumikka no ryokou e iki fuzei aru ryokan ni 【sunde】 nihon no fuyu o tannou shimashita*	1	2	3	4

[ryikan **suku**: live hotel]<br>(I went on a trip with my friend for three days, two nights living (staying) in a hotel and thoroughly enjoyed the Japanese winter)				
30, *maitoshi no chouki yasumi wa sofubo no ie ni* 【*sunde*】 *minna de nigiyaka ni sugoshimasu*<br>[ie **sumu**: live home]<br>(Every year, during the long holidays, I live (stay) in my grandparents home and enjoy my time being lively)	1	2	3	4

Appendix 3. Examples of test sentences for L1-L2 collocations

L1-only				
1, *chaimu ga nattara doa o* 【*akete*】 *kudasai*<br>[doa **akeru**: open door]<br>(Please open the door when the bell rings.)	1 unacceptable	2 slightly-unacceptable	3 slightly-acceptable	4 acceptable
2, *doa o* 【*ake*】*tara bikkuri! Obasann kara no tanjoubi purezento ga haitteimashita*<br>[hako **akeru**: open box]<br>(I was surprised when I opened the box. There was a birthday gift from my aunt in it)	1	2	3	4
3, *otouto wa sakki kara kuchi o* 【*akete*】 *bootto shite iru*<br>[kuchi **akeru**: open mouth]<br>(My brother has been dazed with his mouth open for a while)	1	2	3	4
4, *tobira o* 【*akerut*】 *to suzushii kaze ga haitte kita*<br>[tobira **akeru**: open door]<br>(When I opened the door, a cool breeze came in)	1	2	3	4
5, *pettobotoru no futa o* 【*aketara*】 *awa ga juwatto detekita*<br>[futa **akeru**: open bottle]<br>(I opened the lid of the plastic bottle and the bubbles sizzled out)	1	2	3	4
6, *kouchou sensei ga mon o* 【*akete*】 *seito no toukou o mimamotte imasu*<br>[mon **akeru**: open gate]<br>(The principal opens the gate and watches over the students as they arrive at school)	1	2	3	4
7, *doa o shimetara kanarazu kagi o* 【*akaket*】*e kudasai*<br>[doa **shimeru**: close door]<br>(Please be sure to lock the door after you close the door)	1	2	3	4
8, *mushi ga haitte kuru node natsuba wa mado o* 【*shimet*】*e kudasai*<br>[mado **shimeru**: close window]<br>(Please close the window in the summer because insects come in)	1	2	3	4

9, *Te o arai owattara shikkarito jyaguchi o* 【*shimete*】 *ne*<br>[jyaguchi **shimeru**: turn off faucet]<br>(After washing hands, please securely turn off (close) the faucet)	1	2	3	4
10, *saigo ni kyoushitsu o deru hito wa tobira o* 【*shime*】 *wasurenai youni shitekudasai*<br>[tobira **shimeru**: close door]<br>(Lastly, those of you who can do the classroom, please remember to close the door)	1	2	3	4
11, *hayaku shinaito gakkou no mon o* 【*shime*】 *masu yo*<br>[mon **shimeru**: close gate]<br>(If you don't do it quickly, the school gate will close!)	1	2	3	4
12, *asa wa taitei pan o* 【*tabe*】 *masu*<br>[pan **taberu**: eat bread]<br>(We usually eat bread in the morning)	1	2	3	4
13, *yoru shichiji goro wa daitai gohan o* 【*tabete*】 *iru jikan desu*<br>[gohan **taberu**: eat dinner]<br>(Around seven pm is usually the time we eat dinner)	1	2	3	4
14, *nikudakedenaku yasai mo shikari* 【*tabena*】 *to karada ni yokunai desu*<br>[yasai **taberu**: eat vegetable]<br>(It is not good for your body if you don't eat vegetables but only meat)	1	2	3	4
15, *atsukutemo juusu bakkari* 【*noma*】 *nai youni chuui shimashou*<br>[juusu **nomu**: drink juice]<br>(Let's avoid drinking much juice even it is hot outside)	1	2	3	4
16, *maiasa koohii o ippai nomuto atama ga kasseika* 【*shimasu*】<br>[okayu **nomu**: drink rice gruel]<br>(Drinking a cup of coffee every morning activates your brain)	1	2	3	4
17, *osake o nondara kuruma no unntenn wa shinai you ni* 【*shima*】 *shou nikka desu*<br>[osake **nomu**: drink alcohol]	1	2	3	4

(Don't drive a car after drinking alcohol)				
18, *uchi dewa chichi ba ryouri o* 【*tsukutte*】 *imasu*<br>[ryouri **tsukuru**: make food]<br>(In my family, my dad makes the food)	1	2	3	4
19, *hitorigurashi o shite kara wa jibunnde o benntou o* 【*tsukutte*】 *imasu*<br>[obenntou **tsukuru**: make lunch box]<br>(Since living alone, I make my own lunch box)	1	2	3	4
20, *hoomupeeji o* 【*tsukutte*】 *wagashua no shouhin o senndenn shitai to kanngaete imasu*<br>[hoomupeeji **tsukuru**: make homepage]<br>(We are thinking we would like to advertise our product by creating (make) a home page)	1	2	3	4
21, *ninnki kashu ga sutteji ni* 【*agatta*】 *shunnkann kaizhou wa kannki de ippai ni narimashita*<br>[suteeji **agaru**: go up stage]<br>(The moment the popular singer got on (go up) the stage, the stadium was filled with joy)	1	2	3	4
22, *haiyuu wa butai ni* 【*agari*】 *enngi o shimashita*<br>[butai **agaru**: go up stage]<br>(The actor got on (go up) the stage and began acting)	1	2	3	4
23, kaeru wa ryouseirui nanode suichuu ni irukoto mo riku ni 【*agaru*】 *koto mo dekiru*<br>[riku **agaru**: go up land]<br>(Frogs are amphibians so they can be in the water and also go on (go up) to land)	1	2	3	4
24, *gakkou no okuzhou ni* 【*agaru*】 *to machi o ichibou dekimasu*<br>[okuzhou **agaru**: go up roof]	1	2	3	4

(By going up (go up) on the roof of the school, we can see the view of the town)				
25, *daigakusei no musume wa daigaku no ryou ni* 【*sunnde*】 *imasu*<br>[ryou **sumu**: live dorm]<br>(My daughter who is a college student lives (live) in the college dorms)	1	2	3	4
26, shourai wa *tokai ni* 【*sumitai*】 *ga taikiosenn ya bukkadaka nado ga shinnpai da*<br>[tokai **sumu**: live city]<br>(I would like to live in a city in the future, but I am worries about air pollution and high prices)	1	2	3	4
27, *rougo wat aue nado mo shitai node shizuka ni inaka ni* 【*sumitai*】 *to omotteiru*<br>[yado **sumu**: rural house live]<br>(I would like to live in a quiet countryside because I would like to plant rice in my old age)	1	2	3	4
28, *kotoshi no natsu gaikoku ni* 【*sunnde*】 *iru ane ni aini ikimasu*<br>[gaikoku **sumu**: live abroad]<br>(This summer, I will go to see my older sister who lives (live) abroad)	1	2	3	4
29, *yuujinn wa mannshonn ni* 【*sunnde*】 *iru node ookii inu wa kaenai ga chiisai inu nara kaeru to itteita*<br>[mannshonn **suku**: live apartment]<br>(My friend lives in an apartment, so she can't have a big dog, but she can have a small dog)	1	2	3	4
30, *kaigai ni* 【*sumi*】 *tai to omouga gogakuryoku o motto agenakereba muzukashii kamoshirenai*<br>[kaigai **sumu**: live abroad]<br>(I would like to live abroad, but it may be difficult without improving my language skills)	1	2	3	4
